# Ovariectomy Induces Microglial Cell Activation and Inflammatory Response in Rat Prefrontal Cortices to Accelerate the Chronic Unpredictable Stress-Mediated Anxiety and Depression

**DOI:** 10.1155/2020/3609758

**Published:** 2020-05-16

**Authors:** Fei Ge, Haoran Yang, Weiting Lu, Huilian Shi, Qinlei Chen, Yi Luo, Lina Liu, Jing Yan

**Affiliations:** ^1^Department of Gastroenterology, Haian Hospital of Traditional Chinese Medicine Affiliated to Nanjing University of Chinese Medicine, Haian 226600, China; ^2^Department of Hepatology, The Affiliated Hospital of Nanjing University of Chinese Medicine, Nanjing 210029, China; ^3^First Clinical Medical College, Nanjing University of Chinese Medicine, Nanjing 210023, China; ^4^Department of Oncology, Affiliated Hospital of Integrated Traditional Chinese and Western Medicine, Nanjing University of Chinese Medicine, Nanjing 210028, China; ^5^Key Laboratory for Metabolic Diseases in Chinese Medicine, First Clinical Medical College, Nanjing University of Chinese Medicine, Nanjing 210023, China

## Abstract

Perimenopausal women are associated with increased risks of depression and anxiety, which may be potentially related to the lack of ovarian hormone with antidepression activity in the body. However, the precise mechanism remains unclear so far. This study first adopted the Sprague-Dawley (SD) female rats to construct the ovariectomy (OVX) combined with a chronic unpredictable stress (CUS) model. Then, a series of behavioral experimental results revealed that the ovariectomized rats receiving CUS had remarkably elevated anxiety and depression behaviors relative to those in sham group rats, and the sucrose preference rate in the sucrose preference test (SPT) was evidently reduced. In elevated plus maze test (EPM) experiment, the open arm entry time and open arm duration were decreased. In the open field test (OFT), the number of line crossings, rearing number, center square entries, and center square duration were reduced; the grooming time was extended; and the number of fecal particles in rats was increased. In the forced swimming test (FST), the rat immobility rate was increased, while the numbers of swimming and crawling were decreased. Afterwards, we discovered that OVX downregulated the serum levels of estradiol and corticosterone in rats. Thereafter, IF results suggested that OVX dramatically induced the increasing of the number of activated microglial cells in prefrontal cortices and the level of M1-type marker iNOS. Finally, PCR results demonstrated that, compared with the sham group, the proinflammatory and prooxidative genes, such as IL-1*β*, IL-6, TNF-*α*, iNOS, and CX3CR1, were upregulated in the prefrontal cortices of OVX rats after CUS stimulation, whereas the anti-inflammatory factor Arg1 and microglial cell negative regulatory factor CD200 were downregulated. To sum up, OVX enhances the CUS-mediated anxiety and depression phenomena in rats, and its mechanism may be related to inducing the activation and polarization of microglial cells in the prefrontal cortex of animal and to accelerating the inflammatory response.

## 1. Introduction

The incidence of emotional disorder is different between different genders; generally, women and girls are more likely to develop anxiety or depression than males [1]. Moreover, compared with other age groups, females aged 45-55 years are associated with a higher risk of depression, which suggests that changes in hormone levels in perimenopausal and menopausal transition periods may be the risk factor of the occurrence of depression and anxiety symptoms [2]. Supporting this speculation, a cohort study on ovariectomy (OVX) and senescence carried out in the Mayo Clinic suggests that bilateral OVX in premenopausal women is related to the increased risks of long-term depression and anxiety symptoms in patients [3]. Moreover, data obtained from an animal study show that injection of estradiol (E2) and progesterone in ovariectomized rodents reduces the rest time in the forced swimming test (FST) [4, 5], while increasing the open arm duration of the animals in the elevated plus maze test (EPM), which displays the antidepression effect [6, 7]. In clinical practice, we have currently applied the estrogen replacement therapy (ERT) in treating the depression and anxiety symptoms in perimenopausal women [8–11]. Therefore, ovarian hormone exhibits certain antidepression and antianxiety effects, while long-term ovarian hormone deprivation or OVX is the risk factor of the occurrence of emotional disorder [12]. However, the underlying molecular mechanism remains unclear so far.

Recent research demonstrates that brain tissue inflammation plays a vital role in the genesis and development of emotional disorder, among which the microglial cells within the prefrontal cortices exert a crucial part [13–15]. Microglial cell belongs to the mononuclear phagocyte system, which is a kind of immune cell in the central nervous system (CNS). Under normal condition, microglial cell is at the resting state to maintain the normal physiological functions of the brain. In the presence of multiple stimulating factors (such as pressure stimulation, infection, and bleeding), microglial cell is activated and transformed from the branch shape at resting state to the amoeba shape with phagocytosis; meanwhile, it releases the mixture of cytokines and other inflammatory molecules [16]. The activated microglial cell is classified as M1 and M2 polarized states [17]. The polarized M1 microglial cell produces the proinflammatory cytokines and neurotoxicity to participate in the occurrence of neural network dysfunction and promote inflammation. By contrast, the polarized M2 microglial cell secretes the anti-inflammatory mediators and participates in restoring the balanced neurotrophic factors in the body, which contributes to inflammation resolution and promoting neuron survival. Additionally, some research verifies that change in ovarian hormone level or stimulation of chronic pressure (two key risk factors of depression and anxiety in perimenopausal women) is reported to activate the microglial cells in prefrontal cortices from the resting state to the activated state, induce the polarization into M1 type (markers of M1 type microglial cells: upregulation of CD11b, CD14, CD18, CD45, CD74, CD86, TLR4, and iNOS levels), release a large amount of inflammatory factors, and induce the occurrence of inflammatory response [18, 19]. However, the relationships of microglial cell activation and polarization, as well as the mediated inflammatory response with the depression and anxiety status in perimenopausal women, have not been directly verified in animal experiment or clinical sample, which should be further confirmed.

Pressure stimulation is also a key risk factor for the occurrence of emotional disorder in a perimenopausal period. Therefore, this study first constructed the rat model to verify the accelerating effect of OVX (ovarian hormone deprivation) on the chronic unpredictable stress- (CUS-) mediated depression and anxiety symptoms based on behavioral experimental approaches. Subsequently, the molecular biological means (immunofluorescence (IF) and Q-PCR) were employed to observe the effects of OVX on regulating the activation and polarization of microglial cells in rat prefrontal cortices and the expression of key genes related to inflammatory response. This project contributes to further understanding the molecular mechanisms of the genesis and development of depression and anxiety symptoms among perimenopausal women and determining the core role of microglial cells in prefrontal cortex, which provides novel theoretical foundation for disease treatment.

## 2. Materials and Methods

### 2.1. Experimental Animals and Grouping

48 7-8-week-old female Sprague-Dawley (SD) rats weighing 200 ± 20 g at specific pathogen-free (SPF) grade were provided by Beijing Vital River Laboratory Animal Technology Co., Ltd. Then, all animals were raised under a 12 h/12 h light-dark cycle environment (07:00-19:00), with a room temperature of 25°C and a relative humidity of 50-55%, and the animals had free access to food and water. Prior to the experiment, all animals were allowed to acclimate for 7 days. The study protocol was approved by the Laboratory Animal Institutional Ethics Committee of Nanjing University of Chinese Medicine. The 48 rats were randomly divided into 4 groups (*n* = 12 for each group), including the sham group (CON), sham+CUS group, OVX group, and OVX+CUS group.

### 2.2. Construction of the CUS Model

The experiment was started at 9:00 every morning, and any one of the following stimulation methods was selected every day for 6 weeks continuously. The stimulation projects included cage rocking (5 times per second) for 15 min, swimming in cold (4°C) water for 5 min, soiled cage for 24 h, lights on overnight, cage tilting for 24 h, restraint for 12 h, food and water deprivation for 24 h, elevated temperature (40°C) for 15 min, and electric stimulus (1.0 mA, each time for 1 s, 10 times per minute) for 5 min. Thus, the CUS model was constructed, and the rat pain and discomfort were minimized during the whole stimulation process, so as to maximize the stimulation unpredictability. The animal weights were weighed every week during the CUS period.

### 2.3. Ovariectomy (OVX)

The rats were given intraperitoneal injection of 3% pentobarbital for anesthesia, and the skin was prepared. In the lower 1/3 of the trunk, bilateral longitudinal incisions (about 1 cm in length) were made at 1-2 cm away from the spinal cord. The cellulite was pulled out using the forceps, and a mass of flesh-colored fine-line irregular tissue was found, which was the ovary. It was connected downward with the large tubular tissue, which was the cornua uteri. Then, the ovary was ligated and cut off, together with the remaining line, and the incisions were sutured. For the sham group, fat tissue equivalent to the ovary volume was cut off, and the other operation was the same as the above. Then, the animals were intraperitoneally injected with penicillin once a day for 7 consecutive days. CUS was carried out in rats after 1 week of OVX modeling.

### 2.4. Behavioral Experiments

#### 2.4.1. Sucrose Preference Test (SPT)

Two drinking water bottles filled with 1% sucrose were placed in the animal cages at the same time, and the animals were trained to adapt to the glucose-containing drinking water for 24 h. After food and water deprivation for 8 h, each rat was given a bottle of pure water and a bottle of 1% sucrose-containing water, with consistent appearance and volume. After 24 h, the remaining liquid volumes were measured, respectively; thereafter, the total liquid consumption, sucrose water consumption, and SPT rate of each animal were calculated: SPT rate = (sucrose water consumption/total liquid consumption) × 100%, which represents the degree of anhedonic behavior in animals.

#### 2.4.2. Elevated Plus-Maze Test (EPM)

The elevated plus-maze was 60 cm above the ground level, which was constituted by two opposite open arms (length × width of 40 cm × 14 cm), two opposite enclosed arms (length × width × height of 40 cm × 14 cm × 28 cm), and a central platform to combine the four arms (14 cm × 14 cm). All animals entered the test room at 1 h prior to the experiment. Before the EPM, the rats for the test were placed in a cage and allowed to explore for 5 min before they were placed in the central platform of the EPM, so that their heads were dead against any one of the open arms. The following indexes were monitored and recorded after release (within 5 min): (1) open arm entry (OE), (2) open arm time (OT), (3) close arm entry (CE), and (4) close arm time (CT). Based on the (1)-(4) values, the following items were calculated, respectively: (a) total entry of open arm and close arm (total entry); (b) ratio of OE, namely, OE/(OE + CE) × 100%; and (c) open arm duration ratio, namely, OT/(OT + CT) × 100%. The above-mentioned experiments were analyzed using the animal behavior analysis software (version 1.00, Clever Sys Company, USA).

#### 2.4.3. Open Field Test (OFT)

The OFT experimental apparatus was a cube with a dimension of 60 cm × 60 cm × 60 cm, and the square on the bottom was evenly divided into 16 small squares. The region constituted by 4 small squares (30 cm × 30 cm) close to the center was designed as the central region, while that surrounding the center was assigned as the peripheral region. At the beginning of the test, rats were moved out from the cage and gently put into the peripheral region, with their heads facing the corner. The rat activities within the 10 min test period were recorded, including line crossing, rearing time, center square entry, and center square duration, as well as the number of bowel particles within 10 min.

#### 2.4.4. Forced Swimming Test (FST)

The rats were placed in the single glass cylinder (height, 46 cm; diameter, 20 cm; water temperature 23-25°C; and water depth, 30 cm) for swimming. It was required that the rats could not support themselves through contacting the bottom with their claws or tails. A 15 min preexperiment was carried out on the day before the experiment. At 24 h later, the frequencies of the following behaviors were recorded within 5 min: (1) immobility—floating without struggling and doing only those movements necessary to keep the head above the water; (2) swimming—showing active swimming motions, more than those necessary to merely keep the head above water, namely, moving around in the cylinder or diving; and (3) climbing—presenting active movements with the forepaws, usually directed against the walls.

#### 2.4.5. Detection of Serum E2 and Corticosterone (Cor)

Three days after completing the FST test, the rats were performed thoracotomy under anesthesia with sodium pentobarbital (intraperitoneal injection at 40 mg/kg), along with intubation to the ascending aorta through the left ventricle. The trunk blood was collected using the serum separation tube; the blood sample was allowed to coagulate under room temperature and centrifuged at 1000 g for 15 min at 4°C to separate the serum. Then, the serum E2 and Cor levels were detected using the ELISA kit (Shanghai Enzyme-linked Biotechnology Co. Ltd., Shanghai, China).

#### 2.4.6. Immunofluorescence (IF)

Three rats in each group were given transcardiac perfusion of cold saline and fixed with 4% paraformaldehyde for 1 h; later, the brains were resected and fixed in the 4% paraformaldehyde for 24 h, followed by paraffin embedding. Afterwards, the frontal cortex position (interaural 14.64 mm, bregma 5.64 mm) was selected for paraffin section with reference to the stereotaxic map of a rat brain. After deparaffinage, antigen retrieval was carried out in a retrieval box using the EDTA (pH 8.0) antigen retrieval solution (Servicebio Technology Co., Ltd., Wuhan, China). Then, fluorescence quencher was added, and BSA was also added dropwise for incubation; then, primary antibodies (anti-ionized calcium-binding adapter molecule-1 (anti-Iba-1; Servicebio Technology Co., Ltd., Wuhan, China); anti-rabbit-INOS (1 : 100, Proteintech Group, Inc., USA)) were added to incubate overnight at 4°C, followed by incubation with fluorescence secondary antibodies (goat anti-mouse (Alexa 488 conjugated, 1 : 100, Proteintech Group, Inc., USA); goat anti-rabbit (Alexa 594 conjugated, 1 : 100, Proteintech Group, Inc., USA)) in the dark at room temperature. Later, DAPI (Servicebio Technology Co., Ltd., Wuhan, China) was added to counterstain the cell nuclei, followed by mounting with the antifluorescence quenching mounting medium. The sections were observed under a fluorescence microscope (Nikon Instruments Inc., Mode: NIKON ECLIPSE C1), and images were collected. Each experiment was implemented for at least three times independently, and the positive cell number was measured using Image-Pro Plus 6.0 (Media Cybernetics, Inc.).

#### 2.4.7. Real-Time Quantitative Polymerase Chain Reaction

Six rats in each group were sacrificed, the skull in each animal was dissected, and the brain cortices were rapidly collected and placed on ice. The PFC region was rapidly positioned, sliced into cubes (1 × 1 × 0.5 mm^3^), and immediately placed into the liquid nitrogen for freezing. The tissues were collected before experiment and washed with cold PBS, and the total cellular RNA was extracted from the tissues using TRIzol reagent (Vazyme Biotech Co., Ltd., Nanjing, China). Then, 1 *μ*L total cellular RNA was mixed with 4 *μ*L Prime Script TM Master Mix (Takara Biomedical Technology Beijing Co., Ltd.) and 15 *μ*L RNase-free ddH_2_O (Vazyme Biotech Co., Ltd., Nanjing, China) to prepare the reverse transcription reaction solution for reverse transcription. The specific conditions were as follows: at 37°C for 15 min, at 85°C for 5 s, and at 4°C for 10 min. Finally, 5 *μ*L SYBR Green Mix (article number Q1321-02, Nanjing Nuoweizan Biology), 2 *μ*L cDNA template, and upstream and downstream primers (10 *μ*mol·L^−1^) were added to prepare the mixed solution for Q-PCR assay under the conditions of 1 cycle at 95°C for 5 s, followed by 40 cycles at 95°C for 5 s and at 60°C for 31 s. The melting conditions were shown below, at 95°C for 15 s, at 60°C for 30 s, and at 95°C for 15 s. Eventually, the relative fold change was calculated according to the 2^(-*ΔΔ*Ct)^ method. The primers were synthesized by Shanghai Sangon Bioengineering Co. Ltd. The sequences are displayed in Table 1.

IL-1*β*: interleukin-1*β*; IL-6: interleukin-6; TNF-*α*: tumor necrosis factor; INOS: inducible nitric oxide synthase; ARG1: arginase-1; CD200: the cluster of differentiation 200; CD200R: the receptor of CD200; CX3CL1: the unique member of the CX3C chemokine subfamily; CX3CR1: the receptor of CX3CL1; GAPDH: glyceraldehyde-3-phosphate dehydrogenase.

### 2.5. Statistical Analysis

Data were expresses as the mean ± SEM. The SPSS version 18.0 (SPSS Inc.) was adopted for data analysis. If the data conformed to the homogeneity of variance in the analysis of variance, the significance of differences between groups was evaluated by one-way analysis of variance (ANOVA) followed by the LSD test; otherwise, it was analyzed by the pairwise comparisons in the Kruskal-Wallis test. *P* < 0.05 was considered to indicate a statistically significant difference.

## 3. Results

### 3.1. OVX Markedly Deteriorated the Anxiety and Depression Status of Rats Mediated by CUS

**Figure 1:**
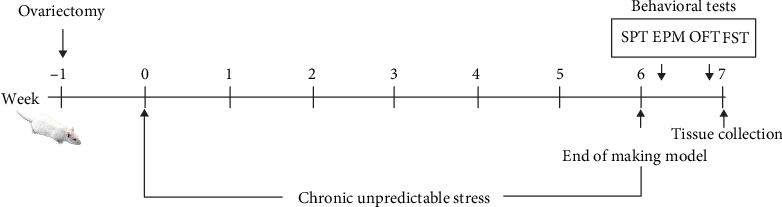
Experimental protocol. Behavioral tests were performed after 6 weeks of chronic unpredictable stress and 7 weeks of ovariectomy in the female rats. SPT: sucrose preference test; EPM: elevated plus-maze test; OFT: open field test; FST: forced swimming test.

**Figure 2:**
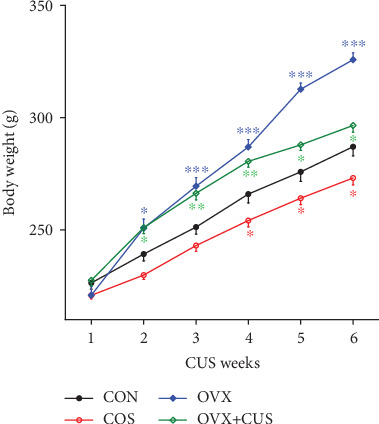
The body weight of four groups of ovariectomy and/or unpredictable stress rats during 6 weeks of chronic stress. Data are presented as the means ± SEM (*n* = 10 rats per group). Compared the weight with the control rats of the same age. ^∗^*P* < 0.05; ^∗∗^*P* < 0.01; ^∗∗∗^*P* < 0.001. CON: control group; CUS: chronic unpredictable stress group; OVX: ovariectomy group; OVX+CUS: ovariectomy combined with chronic unpredictable stress group.

**Figure 3:**
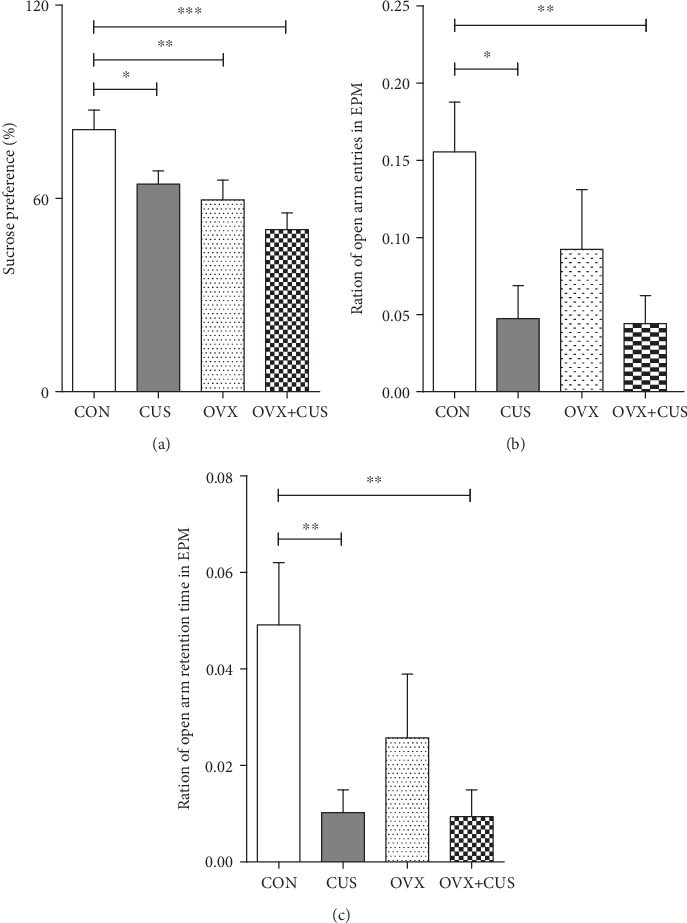
Effects of ovariectomy and/or chronic unpredictable stress on behavioral changes in the sucrose preference test and elevated plus-maze test in the female rats. (a) Sucrose uptake rate in the sucrose preference test. (b) Ratio of open arm entering times in the elevated plus-maze test. (c) Ratio of open arm retention time in the elevated plus-maze test. Data are presented as the means ± SEM (*n* = 10 rats per group). ^∗^*P* < 0.05; ^∗∗^*P* < 0.01; ^∗∗∗^*P* < 0.001. CON: control group; CUS: chronic unpredictable stress group; OVX: ovariectomy group; OVX+CUS: ovariectomy combined with chronic unpredictable stress group.

**Figure 4:**
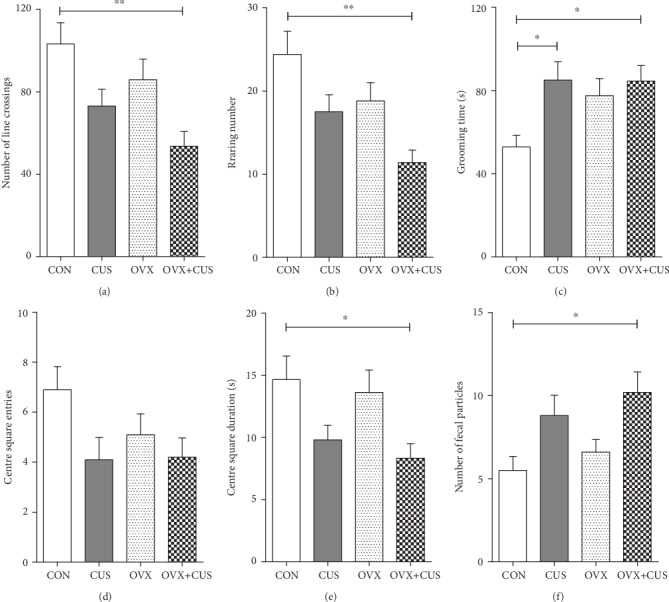
Behavioral of open field test was conducted, and the excretion of stool was checked on ovariectomy and/or chronic unpredictable stress rats. (a) Number of line crossings. (b) Rearing number. (c) Grooming time. (d) Center square entries. (e) Center square duration. (f) Number of fecal particles. Data are presented as the means ± SEM (*n* = 10 per group). ^∗^*P* < 0.05; ^∗∗^*P* < 0.01; ^∗∗∗^*P* < 0.001. CON: control group; CUS: chronic unpredictable stress group; OVX: ovariectomy group; OVX+CUS: ovariectomy combined with chronic unpredictable stress group.

**Figure 5:**
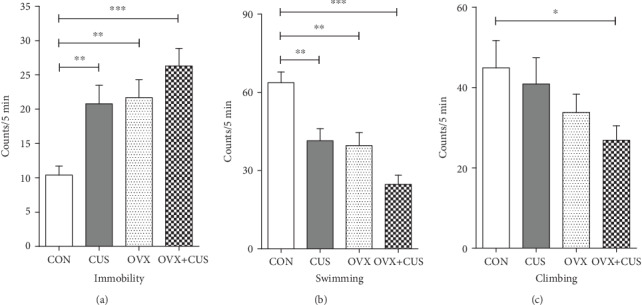
Effects of ovariectomy and/or chronic unpredictable stress on the levels of (a) immobility, (b) swimming, and (c) climbing behaviors of the female rats in the forced swimming test during a 5 min test period. Data are presented as the means ± SEM (*n* = 10 per group). ^∗^*P* < 0.05; ^∗∗^*P* < 0.01; ^∗∗∗^*P* < 0.001. CON: control group; CUS: chronic unpredictable stress group; OVX: ovariectomy group; OVX+CUS: ovariectomy combined with chronic unpredictable stress group.

In this part, the animal models were constructed for different groups, as shown in the mode pattern in Figure 1. After 6 consecutive weeks of CUS stimulation, multiple behavioral experiments were applied to evaluate the anxiety and depression status of rats in each group. First, it was observed from the statistical results on the average body weight of each group (Figure 2) that the weights of animals receiving OVX (OVX group and OVX+CUS group) were higher than that in the sham group due to ovarian hormone deprivation. Besides, CUS markedly reduced the weights of animals receiving OVX or not (CON vs CUS, OVX+CUS vs OVX). The subsequent SPT (Figure 3(a)) suggested that, compared with the CON group, the SPT rates in the other three groups were evidently reduced, among which the OVX+CUS group suffered from the most obvious decline. For the two single stimuli, OVX better suppressed the sucrose preference in animals than CUS. Similarly, in the EPM (Figures 3(b) and 3(c)), the open arm entries and open arm duration in the other three groups were markedly lower than those in the CON group. With regard to the two single stimuli, CUS better suppressed the animal mobility than OVX, while the combination of these two stimuli (CUS+OVX) did not further suppress the exploratory activities of rats. Subsequent OFT experimental results (Figure 4) suggested that, compared with those in the CON group, the number of line crossings, rearing number, center square entries, and center square duration in the other three groups were dramatically reduced, while the grooming time was evidently extended and the number of fecal particles was remarkably increase. Compared with CUS or OVX single stimulation, the dual stimuli of OVX+CUS resulted in higher depression and anxiety levels in that behavioral experiment, along with a relatively less number of line crossings, rearing number, and center square duration, with a relatively higher number of fecal particles. Results of the eventual FST (Figure 5) demonstrated that, compared with that in the CON group, the immobility of animals in OVX+CUS group was increased at the highest level, with the most obvious decreases in swimming and climbing times. The above behavioral results manifested that OVX markedly aggravated the CUS-mediated anxiety and depression in rats.

**Figure 6:**
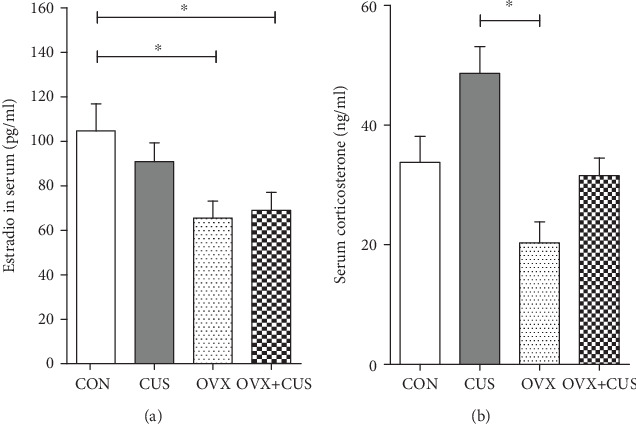
Effects of ovariectomy and/or chronic unpredictable stress on the (a) serum estradiol and (b) serum corticosterone levels of female rats. Data are presented as the means ± SEM (*n* = 10 per group). ^∗^*P* < 0.05. CON: control group; CUS: chronic unpredictable stress group; OVX: ovariectomy group; OVX+CUS: ovariectomy combined with chronic unpredictable stress group.

Eventually, the serum estrogen and corticosterone levels in each group were detected using the ELISA method. The results are presented in Figure 6. Compared with non-OVX rats (CON and CUS groups), the serum estrogen and corticosterone levels in animals receiving OVX notably declined. Serum corticosterone is the most well-known characteristic endocrine marker of the hypothalamus-pituitary-adrenal gland (HPA) axis (the key depression regulatory mechanism) [20], which is generally evidently upregulated under the stimulation of CUS. The corticosterone levels shown in Figure 6 suggested that, relative to the mechanism of CUS modeling alone, the mechanism of combined OVX+CUS modeling in further inducing animal activity suppression, weakened exploratory capacity, and chronic stress-induced anhedonia might not be related to the traditional HPA axis, and the novel molecular foundation should be further explored.

### 3.2. OVX Markedly Aggravated CUS-Mediated Activation and Polarization of Microglial Cells in Prefrontal Cortex, as Well as the Expression of Inflammatory Factors

**Figure 7:**
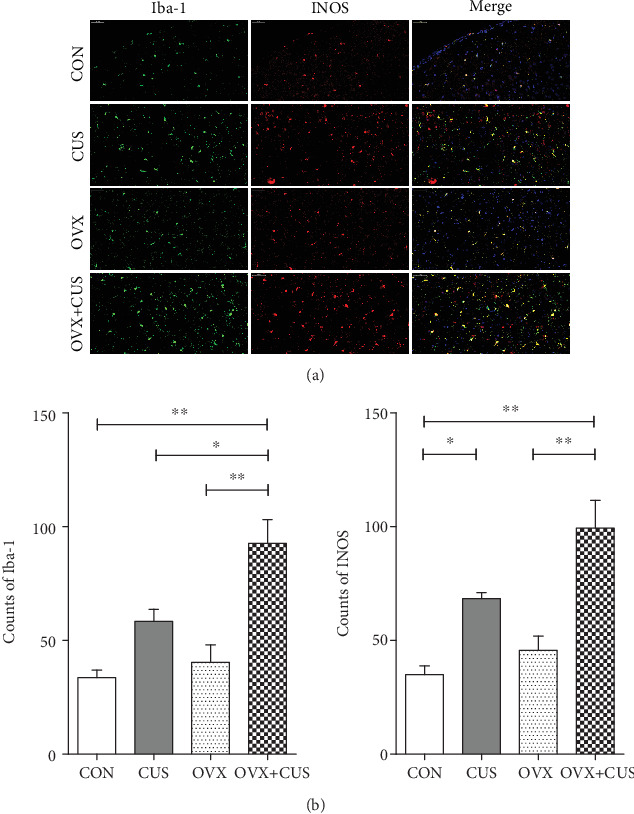
Effects of ovariectomy and/or chronic unpredictable stress on Iba-1 and iNOS in the prefrontal cortex of female rats. (a) Fluorescent double staining of Iba-1 and INOS (×400). (b) Quantitative comparison of positive cells of Iba-1 and INOS. Data are presented as the means ± SEM (*n* = 3 per group). ^∗^*P* < 0.05; ^∗∗^*P* < 0.01; ^∗∗∗^*P* < 0.001. CON: control group; CUS: chronic unpredictable stress group; OVX: ovariectomy group; OVX+CUS: ovariectomy combined with chronic unpredictable stress group.

**Figure 8:**
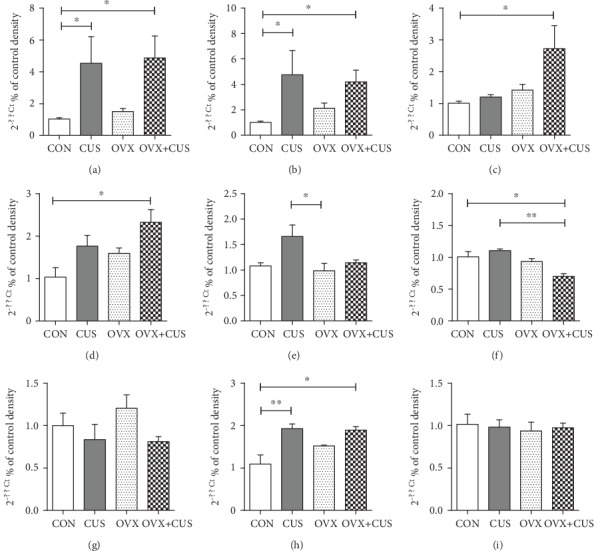
Effects of ovariectomy and/or chronic unpredictable stress on mRNA level of cytokines and the relative genes in the prefrontal cortex of female rats. (a) IL-1*β*, (b) IL-6, (c)TNF-*α*, (d) INOS, (e) Arg1, (f) CD200, (g) CD200R, (h) CX3CR1, and (i) CX3CL1. Data are presented as the means ± SEM (*n* = 6 per group). ^∗^*P* < 0.05; ^∗∗^*P* < 0.01; ^∗∗∗^*P* < 0.001. CON: control group; CUS: chronic unpredictable stress group; OVX: ovariectomy group; OVX+CUS: ovariectomy combined with chronic unpredictable stress group.

Iba-1 is a cytoskeleton protein specific not only for microglia but also for monocyte/macrophage lineage, where it acts as an actin crosslinking protein. But Iba-1 is not a simple cytoskeleton protein, but a signaling molecule involved in specific signaling pathways frequently, and is regarded as a marker of activation [21]. Existing research results suggest that either OVX or CUS affects the activation and polarization of cortical microglial cells. Therefore, we speculated that the effect of OVX+CUS on inducing rat depression and anxiety status might be associated with the regulation of microglial cell functions. To verify this speculation, we first collected the prefrontal cortices from animals for the IF test. The results suggested that (Figure 7), compared with the CON group, the numbers of activated microglial cells (Iba-1^+^) in prefrontal cortices of three groups were markedly elevated and the levels of M1 type marker (iNOS) were also evidently improved. Noteworthy, compared with CUS stimulation alone, combination with OVX (OVX+CUS) could further promote the activation and polarization of microglial cells in rat prefrontal cortices. It is well known that the M1 type microglial cells in brain mainly mediate the intracellular inflammatory response through producing the proinflammatory cytokines, thus participating in the genesis and development of neural network dysfunction. Consequently, we further detected the expression levels of various proinflammatory cytokines in prefrontal cortices. Q-PCR results revealed that (Figure 8), compared with single modeling of OVX and CUS, the combined stimulation of OVX+CUS maximally induced the levels of inflammatory factors in prefrontal cortices, including IL-1*β*, IL-6, and TNF-*α* (Figures 8(a)–8(c)). Thereafter, we detected the expression levels of iNOS and Arg1 in prefrontal cortices. Typically, iNOS and Arg1 are the indicators of oxidative stress/potential proinflammatory and anti-inflammatory functions in microglial cells [22]. It should be noted that iNOS and Arg1 compete for the same amino acid (L-arginine) and, thus, Arg1 regulates the function of iNOS by acting on this substrate, a direct precursor to the synthesis of nitric oxide [23]. As observed from the detection results (Figures 8(d) and 8(e)), the iNOS level in the prefrontal cortices of the OVX+CUS group was markedly upregulated, while the Arg1 level showed no obvious difference compared with that in the sham group, demonstrating that the combined stimulation of OVX and CUS evidently induced the occurrence of intracellular inflammation and oxidative stress and resulted in neurotoxicity. Such results were consistent with the previous results on the proinflammatory cytokine expression levels. Moreover, we also examined the expression levels of CD200 and its receptor CD200R in prefrontal cortices. CD200R is generally expressed on the microglial cell membrane; the combination of neuronal and astroglial CD200 with the microglial CD200R in prefrontal cortices regulates microglial surveillance, inhibits microglial activation, and reduces proinflammatory cytokine expression [24, 25]. As shown in Figures 8(f) and 8(g), the CD200 and CD200R levels in the prefrontal cortices of the OVX+CUS group were evidently downregulated compared with those in other groups, revealing the high activation status of microglial cells inside. Finally, the chemokine, fractalkine (CX3CL1), and its cognate receptor (CX3CR1) were analyzed. CX3CR1 is generally expressed on the surface of microglial cell membrane, which binds with CX3CL1 within the tissue space to induce microglial cell activation, recruit a large amount of immune cells, and mediate a series of inflammatory responses. Some reports suggest that hampering neuron-microglia communication via the CX3CR1-CX3CL1 pathway prevents the effects of CUS on microglial function, as well as the short- and long-term neuronal plasticity and depression-like behavior [26, 27]. As observed from Figures 8(h) and 8(i), OVX+CUS remarkably induced CX3CR1 expression in rat prefrontal cortices, but it made no distinct difference to the CX3CL1 level. To sum up, compared with the sham and CUS single stimulation groups, the dual stimuli of OVX+CUS more evidently induced the expression of proinflammatory genes (IL-1*β*, IL-6, and TNF-*α*) and oxidative stress factor (iNOS) in prefrontal cortices. The underlying mechanisms might be related to inducing the activation (suppressing the CD200-CD200R interaction, upregulated the level of CX3CR1) and M1 polarization (iNOS upregulation and Arg1 downregulation) of microglial cells.

## 4. Discussion

Perimenopausal emotional disorder refers to a class of mood disordered mental illness that first occurs during the perimenopausal period and characterized by emotional disorder, retardation of thinking, and cognitive decline [28]. Research suggests that the pathogenesis of emotional disorder is related to sex, and the incidence in female is about twice of that in male. Female emotional behaviors are affected by the fluctuations of gonadal hormone levels in the body, and women are likely to develop emotional disorder at special time points of hormone changes, such as premenstrual period, gestation period, and puerperium and perimenopausal period. Typically, the incidence of some disorder peaks at the perimenopausal period [29, 30]. It has been verified that stress is the major external cause of the incidence of perimenopausal emotional disorder, whereas ovarian hormone deprivation (internal cause) is also closely related to disease occurrence. With the increasingly elevated social living pressure [31, 32], the incidence of perimenopausal emotional disorder shows a growing trend year by year, which has gradually become the public health and social problem that severely affects female physical and mental health, as well as family harmony [33]. A clinical research discovers that perimenopausal women suffer from decline in ovarian function, and the high morbidity of perimenopausal depression (PMD) is related to the low circulating estrogen level, and changes in ovarian estradiol secretion can trigger the abnormal mental behavior in women [34].

Ovarian hormone, the natural neuroprotective factor, is a group of steroidal hormone that has neuroregulation and neuroprotective effects. Research suggests that estrogen can affect neurotransmitter synthesis, neuron growth, synapse formation, neuronal spine density, and neural conduction. In addition, it regulates the growth of astrocytes and microglial cells, promotes nerve fiber formation, and suppresses neuron apoptosis [35, 36]. At the same time, estrogen receptor is distributed in numerous anxiety- and depression-related regions, and estrogen can cross the blood brain barrier to affect multiple emotion-associated neurotransmitter systems through the estrogen receptor; for instance, 5-hydroxytryptamine, dopamine, and norepinephrine improve the emotional and mental disorders [37].

As a result, it regulates the central nervous system (CNS) structure and function, modulates emotional reactions and cognitive function, and exhibits the neuroprotective effect. Consequently, estrogen replacement therapy (ERT) or estrogen combined with an antidepressant is mostly adopted in clinic to treat perimenopausal emotional disorder at present [38–40]. Such method has a definite therapeutic effect, but it is associated with numerous contraindications of long-term hormone application and potential carcinogenicity [41]. The discovery of small molecule compound with a reliable efficacy and low side effect is of vital importance to the clinical treatment for perimenopausal emotional disorder. First of all, we should determine the role of ovarian hormone deprivation in the genesis and development of perimenopausal emotional disorder, as well as the potential pathological molecular mechanism, so as to develop the novel therapeutic target and the small molecule drugs.

The successful preparation of the perimenopausal emotional disorder animal model is the essential condition to simulate the clinical patient symptoms and manifestations, thus investigating its etiology, pathogenesis, and developing drugs [42]. Perimenopausal emotional disorder is associated with physiological specificity and pathological complexity; as a result, the construction of animal model of this disease in scientific research is partially different from the simple emotional disorder (depression or anxiety). In this research, the perimenopausal emotional disorder rat model was prepared through OVX combined with CUS, based on the theoretical foundation that the declined ovarian hormone level in females and the external environmental stimulation were regarded as the two major internal and external factors responsible for the onset of perimenopausal emotional disorder. This method maximally simulates the core symptoms and similar etiology of perimenopausal emotional disorder, with potent maneuverability [43]. Moreover, OVX completely simulated the ovarian hormone deprivation status in the body, so this model provided congenital advantages to determine the role of ovarian hormone deprivation in the genesis and development of perimenopausal emotional disorder.

Animal behavioral change is a symbol to evaluate whether the emotional disorder model is successfully constructed, which also lays the foundation to investigate the role of ovarian hormone deprivation in the genesis and development of perimenopausal emotional disorder and to examine the downstream pathological molecular mechanism in this study. This study tested the mobility scores of rats through OFT and EPM, so as to judge the spontaneous activities and exploratory behaviors of animals. Meanwhile, the swimming immobility time and sucrose preference rate were tested through FST and SPT, so as to assess the degrees of behavioral desperation and anhedonia in animals. According to our results, OVX and CUS induced anxiety- or depression-like behaviors in rats to various degrees, which manifested as reduced animal spontaneous activities and exploratory behaviors, behavioral desperation, and anhedonia. At the same time, modeling using OVX combined with CUS led to more severe anxiety- or depression-like behaviors in rats, revealing that OVX (ovarian hormone deficiency or deprivation) markedly aggravated the CUS-mediated anxiety and depression status in rats, which further determined the key role of ovarian hormone deprivation in the genesis and development of perimenopausal emotional disorder. Meanwhile, PFC is the region that matures at last and decline at first, and it is extremely sensitive to stress [44]. Therefore, PFC was selected as the target intracerebral regions of the study.

It is well known that reactive upregulation of glucocorticoid (GC) (it is corticosterone (CORT) in rats) helps the body to fight against the external stimuli. The persistently elevating GC level will have an adverse effect. As observed during the CUS process, female rats first had high total CORT levels, but the CORT level gradually recovered to baseline level at 2 weeks later [45]. In this study, female rats had depression-like behaviors after 6 weeks of CUS modeling, the serum CORT level in the CUS group showed an increasing trend compared with that in the control group, but the difference was not statistically significant. By contrast, the CORT level in the OVX+CUS group showed a decreasing trend, but the difference was not statistically significant compared with control group. On the one hand, the CORT variation trend indicated that OVX weakens the responsiveness of the HPA axis to CUS. On the other hand, compared with previous studies on male rats, our findings indicated that the mechanism of depression-like behaviors in female rats under CUS was not closely correlated with the CORT level in the HPA axis. This is because estrogen exerts a protective effect and will affect the effect of CORT on pressure [45, 46]. For rats with intact ovary, the estrogen level decreases under chronic stress condition [45], thus relieving its protective effect on female animals, because estrogen can upregulate the density of glucocorticoid receptor (GRs) [47] to resist the stress-induced inflammation and improve the anxiety and depression in female [48]. In this study, the E2 level did not significantly decline in the CUS group, but a decreasing trend was observed. After the combination of CUS with OVX, serum E2 level persistently declined. On the one hand, the downregulated GRs density in central microglial cells promoted the persistent activation of microglial cells [49]. On the other hand, repeated exposure to chronic stress reduced the corticosteroid binding globulin (CBG) level in female plasma [50]. The latter can decrease GR activation to promote inflammation [45].

Microglial cells, which exert the immunological effect, are closely correlated with the occurrence of anxiety and depression status. Microglial cells can induce the occurrence of emotional disorder through various inflammatory mechanisms and noninflammatory mechanisms. However, their correlation with perimenopausal emotional disorder has not been reported yet, which represents a brand new field. The two key factors of perimenopausal emotional disorder, namely, chronic stress and ovarian hormone deficiency, have been verified to regulate the activation and polarization of microglial cells. Consequently, we speculated that the aggravated effect of OVX on CUS-mediated emotional disorder might be related to affecting the microglial cell status. Research on adult rodents indicates that, under stress condition, microglial cells are more sensitive to gonadal hormone, and the stress hormone (GC) mediates partial but not all differences in stress response, and changes in microglial cell morphology are sex-dependent [44]. In research on adult rats, the long-term chronic (over 21 days) stress reduced the intracerebral microglial cell complexity in female animals [51, 52], but there is little research on intracerebral microglial cell activation and cytokine production in female animals. We applied IF approach to confirm that, compared with the control group, after 6 weeks of CUS stress in adult female rats, the microglial cell numbers located in PFC (namely, the Iba-1 expression quantity) did not increase, while the iNOS level elevated. In other words, CUS promoted M1 polarization activation of microglial cells and increased the proinflammatory factors. It is suggested that OVX aggravated microglial cell activation and M1 polarization. Subsequently, PCR was used to confirm that the dual stimuli of OVX combined with CUS markedly induced the expression of proinflammatory cytokines (IL-1*β*, IL-6, and TNF-*α*) and oxidative stress factor (iNOS) in the prefrontal cortices. Its upstream mechanism might be related to inducing microglial cell activation (upregulation of CX3CR1 and suppression of CD200-CD200R interaction) and M1 polarization (upregulation of iNOS level and downregulation of Arg1 level).

To sum up, this study determines that OVX (or ovarian hormone deprivation) enhances the induction of CUS on the depression and anxiety behaviors in the body. The possible mechanisms may be related to inducing the activation and polarization of microglial cells in the prefrontal cortex and accelerating the inflammatory response. It is discovered that chronic stress activates microglial cells to indirectly mediate the proinflammatory effect of NF-*κ*B on microglial cells [49]. NF-*κ*B activation further promotes the synthesis of proinflammatory cytokines, like TNF-*α* [53], and activates the expression of iNOS in the prefrontal cortex, which is related to the stress-induced neuronal injury [54].

## 5. Conclusions

To sum up, this study determines that OVX (or ovarian hormone deprivation) enhances the induction of CUS on the depression and anxiety behaviors in the body. The possible mechanisms may be related to inducing the activation and polarization of microglial cells in the prefrontal cortex and accelerating the inflammatory response.

## Figures and Tables

**Table 1:** Primer sequence of PCR assay.

Gene names	Primer sequence
IL-1*β*	F-GCACAGTTCCCCAACTGGTA
	R-TGTCCCGACCATTGCTGTTT

IL-6	F-CTGGTCTTCTGGAGTTCCGT
	R-TGCTCTGAATGACTCTGGCT

TNF-*α*	F-GGCATGGATCTCAAAGACAACC
	R-AAATCGGACGGTGTGG

INOS	F-GGAGCAGGTTGAGGATTACTTC
	R-AAAAGACCGCACCGAAGAT

ARG1	F-AAGACAGGGCTACTTTCAGGAC
	R-ACCTTCCCGTTTCGTTCCAA

CD200	F-TGTTCCGCTGATTGTTGGC
	R-ATGGACACATTACGGTTGCC

CD200R	F-TGCCAAAATCGGGAGCTA
	R-AGCTAGCATACGGCTGCATT

CX3CR1	F-AGCTGCTCAGGACCTCACCAT
	R-GTTGTGGAGGCCCTCATGGCTGAT

CX3CL1	F-GAATTCCTGGCGGGTCAGCACCTCGGCATA
	R-AAGCTTTTACAGGGCAGCGGTCTGGTGGT

GAPDH	F-GCGAGATCCCGCTAACATCA
	R-CTCGTGGTTCACACCCATCA

## Data Availability

The datasets used and/or analysed during the current study are available from the corresponding author on reasonable request.
